# Impact of Adherence to Guideline-Recommended Surgical Timing on Outcomes in Infective Endocarditis

**DOI:** 10.3390/jcm15145421

**Published:** 2026-07-10

**Authors:** Daniel Pastor-Wulf, Rafael Gonzalez-Manzanares, Jorge Perea-Armijo, Manuel Crespín-Crespín, José López-Aguilera, Manuel Pan, Juan Carlos Castillo, Manuel Anguita

**Affiliations:** 1 Hospital Universitario de Poniente (Poniente University Hospital), 04700 Almería, Spain; 2 Departamento de Medicina (Department of Medicine), Universidad de Córdoba (University of Cordoba), 14071 Córdoba, Spain; 3 Hospital Universitario Reina Sofía (Reina Sofía University Hospital), 14004 Córdoba, Spain; 4 Instituto Maimónides de Investigación Biomédica de Córdoba (Maimónides Institute for Biomedical Research in Córdoba), 14004 Córdoba, Spain; 5 Centro de Investigación Biomédica en Red de Enfermedades Cardiovasculares (CIBERCV), Instituto de Salud Carlos III, 28029 Madrid, Spain

**Keywords:** infective endocarditis, surgical timing, cardiac surgery, mortality, guideline adherence, early surgery

## Abstract

**Background**: Infective endocarditis (IE) frequently requires surgical intervention; however, the prognostic impact of adherence to guideline-recommended surgical timing remains uncertain. We aimed to evaluate the association between adherence to recommended surgical timing and short- and mid-term mortality in patients with IE undergoing surgery. **Methods**: We conducted a retrospective, observational single-center study including consecutive patients diagnosed with IE and an indication for surgery who underwent surgery during the index hospitalization between January 2000 and April 2021. Patients were classified according to whether surgery was performed within or outside the guideline-recommended time frame based on surgical priority. The primary endpoint was 30-day all-cause mortality. Secondary endpoints included in-hospital complications and one-year mortality. Multivariable logistic and Cox regression analyses were performed to evaluate the association between adherence to recommended surgical timing and outcomes. **Results**: Among 368 patients with IE, 282 (76.6%) had an indication for surgery; 193 surgically treated patients were included in the analysis. Surgery was performed within the recommended time frame in 148 patients (76.7%) and outside the recommended time frame in 45 (23.3%). Thirty-day mortality was 20.2%, with no significant differences between groups (19.6% vs. 22.2%; OR 0.85, 95% CI 0.38–1.92; *p* = 0.701). In multivariable analysis, no association was observed between adherence to guideline-recommended surgical timing and 30-day mortality (OR 1.97, 95% CI 0.77–5.04; *p* = 0.159) or one-year mortality (HR 1.19, 95% CI 0.65–2.17; *p* = 0.578). Society of Thoracic Surgeons (STS) score remained independently associated with both short- and mid-term mortality. Results were consistent in a sensitivity analysis including patients who died before surgery and in a propensity-score overlap weighting analysis. **Conclusions**: In this cohort of patients with IE and surgical indication who underwent surgery during the index hospitalization, adherence to guideline-recommended surgical timing was not associated with short- and mid-term mortality. Preoperative risk was independently associated with both short- and mid-term mortality, whereas surgical priority was associated with short-term mortality. Future prospective studies are needed to better characterize the role of surgical timing in the prognosis of IE.

## 1. Introduction

Infective endocarditis (IE) is an infection of the cardiac endothelium, with an annual incidence of 3–10 cases per 100,000 persons, associated with high in-hospital mortality (15–30% according to different series) [1,2,3,4]. Although the cornerstone of treatment is targeted antibiotic therapy for infection eradication, surgery may be required to treat valvular destruction, uncontrolled infection, or paravalvular extension [5]. Accordingly, the European Society of Cardiology (ESC) Guidelines for the management of endocarditis establish three main indications for surgery during the acute phase: the development or worsening of heart failure (HF), uncontrolled infection, and the prevention of systemic embolization [6].

Approximately 50% of patients with IE undergo surgical intervention as part of the treatment algorithm [7], but the optimal timing of surgery remains unknown. Although early surgery may potentially prevent death and severe complications, surgical intervention during the acute phase of IE carries a significant risk for these patients [8]. The decision to operate is complex because it requires balancing the expected benefit against surgical risk, making multidisciplinary assessment essential to ensure appropriate (and not necessarily more frequent) use of surgery, leading to lower mortality [9].

Current European guidelines establish three categories of surgical priority: emergent (within the first 24 h after surgical indication, regardless of the duration of previous antibiotic treatment), urgent (within 3–5 days after indication), and elective (during the same hospital admission) [6]. However, these recommendations are mainly based on previous observational studies evaluating the safety of early surgery (generally defined as surgery during the index hospitalization) and on expert consensus [8,10,11,12,13].

Although several studies have evaluated the safety of early surgery, as well as outcomes according to whether patients with a surgical indication undergo surgery [12,14,15,16], there is still limited evidence assessing whether adherence to guideline-recommended surgical timing is associated with outcomes in patients with IE.

The aim of this study was to analyze, in a cohort of patients diagnosed with IE with a surgical indication who underwent surgery during the index hospitalization, the association between adherence to recommended surgical timing and short- and mid-term outcomes.

## 2. Materials and Methods

### 2.1. Study Design and Participants

We conducted an observational, longitudinal, and retrospective study, in which we analyzed a cohort of patients diagnosed with and/or treated for IE in the Unit of Cardiology of Reina Sofía University Hospital of Córdoba (Spain) between January 2000 and April 2021. Inclusion criteria were patients with a possible or definite diagnosis of IE involving native or prosthetic valves, right- and/or left-sided cardiac structures, or cardiac implantable electronic devices (CIEDs), who had a surgical indication and underwent surgery during the index hospitalization. Surgical indications were classified according to the guideline recommendations applicable at the time of diagnosis and included HF, uncontrolled infection, prevention of embolic events, CIED-related IE, and other indications individually assessed by the Endocarditis Team.

Exclusion criteria were patients with initially suspected IE in whom an alternative diagnosis was ultimately established, patients without a surgical indication, and patients with a surgical indication in whom surgery was not performed because of prohibitive operative risk, a decision not to operate after multidisciplinary assessment by the Endocarditis Team, death before surgery, or clinical improvement leading to revocation of the initial surgical indication.

Patients were managed according to the clinical practice guidelines in force at the time of diagnosis, including the 1998 American Heart Association (AHA) guidelines [17] for patients diagnosed before 2004, and the 2004, 2009, and 2015 ESC guidelines [18,19,20] for patients diagnosed between 2004 and 2009, 2009 and 2015, and after 2015, respectively.

Patients were followed until death or until February 2024, through review of electronic medical records or with telephone contact. The study was approved by the Ethics Committee of Cordoba (Spain) under reference number 5823. The investigation followed the good clinical practice standards and complied with the ethical principles of the Declaration of Helsinki and its subsequent updates.

### 2.2. Definitions

The diagnosis of IE was established according to the guideline-recommended diagnostic criteria applicable at the time of diagnosis [17,18,19,20]. In summary, at least two sets of blood cultures were obtained before initiating treatment (and if negative, additional serological studies and specific culture techniques were performed), and cardiac imaging tests were performed (echocardiography, and when necessary, computed tomography and/or 18F-FDG positron emission tomography).

Although patients were clinically managed according to the guidelines in force at the time of diagnosis, as described above, for the purposes of the present study, surgical indications and timing categories were retrospectively reviewed and harmonized using the 2015 ESC guidelines as the common reference framework [20]. Surgical priority was classified as emergent (<24 h), urgent (<7 days), and elective (within the index hospitalization). We also confirmed that each patient fulfilled the surgical indication criteria established by the guidelines in force at the time of diagnosis. Patients were subsequently classified according to adherence to the guideline-recommended surgical timing as operated within the recommended time frame or outside the recommended time frame. Patients with a surgical indication who died before undergoing surgery were included in an independent sensitivity analysis.

We registered the following outcomes during the index hospitalization: (a) IE-related complications before surgery, including HF, persistent infection and systemic embolism; (b) major adverse cardiovascular events (MACE) before surgery, defined as cerebrovascular accident (CVA), intracranial hemorrhage, major bleeding requiring blood transfusion, acute myocardial infarction (AMI), shock (cardiogenic or non-cardiogenic), pulmonary thromboembolism (PTE) and cardiac arrest (CA) due to ventricular tachycardia (VT) or ventricular fibrillation (VF); and (c) postoperative complications, including CA (due to VT or VF), shock (cardiogenic, septic, haemorrhagic, distributive), major bleeding requiring blood transfusion, HF, CVA and PTE [21].

### 2.3. Endpoints

The primary endpoint was all-cause mortality within the first 30 days after diagnosis. The secondary endpoints were in-hospital complications and 1-year mortality.

### 2.4. Statistical Analysis

A descriptive analysis of baseline characteristics of the cohort was performed, calculating absolute and relative frequencies for qualitative variables and arithmetic mean ± standard deviation for quantitative variables with normal distribution and median (interquartile range (IQR)) for quantitative variables with non-normal distribution. The normal distribution for quantitative variables was determined with the Kolmogorov–Smirnov test and Q-Q plots.

For comparisons between groups, we performed the appropriate parametric and non-parametric tests: Student’s *t*-test or the Mann–Whitney U test for quantitative variables, depending on data distribution, and chi-square test for qualitative variables. We conducted univariable and multivariable logistic regression models to evaluate the association between surgery within the recommended time frame and 30-day mortality. We performed a sensitivity analysis including patients who died before surgery in the outside recommended time frame. One-year mortality between groups was assessed using Kaplan–Meier survival curves, and the association with surgery within the recommended time frame was evaluated with univariable and multivariable Cox models. Variables included in multivariable models were selected a priori based on clinical relevance and the previous literature. To further address confounding by indication, we conducted a sensitivity analysis using propensity score weighting. The propensity score, defined as the probability of surgery within the recommended time frame, was estimated by logistic regression on the same covariates used in the multivariable models (STS score, echocardiographic abscess, and surgical priority). Because no patient classified in the outside-recommended-time-frame group underwent elective surgery, we used overlap weights, which weight each patient by their probability of belonging to the opposite group and estimate the treatment effect in the subpopulation with clinical equipoise. Covariate balance was assessed by standardized mean differences (threshold 0.10), and weighted Cox proportional-hazards models were fitted with robust (sandwich) variance estimators. All the tests were two-sided, and *p*-values < 0.05 were considered statistically significant. No a priori sample size calculation was performed, as this was a retrospective cohort study including all consecutive patients who met the eligibility criteria during the predefined study period. Data were collected, processed, and analyzed with IBM SPSS Statistics version 25.0 for Windows (IBM Corp., Armonk, NY, USA), except for the propensity score weighting analysis, which was performed using R version 4.4.2 (R Foundation for Statistical Computing, Vienna, Austria).

## 3. Results

### 3.1. Patients’ Characteristics

**Figure 1:**
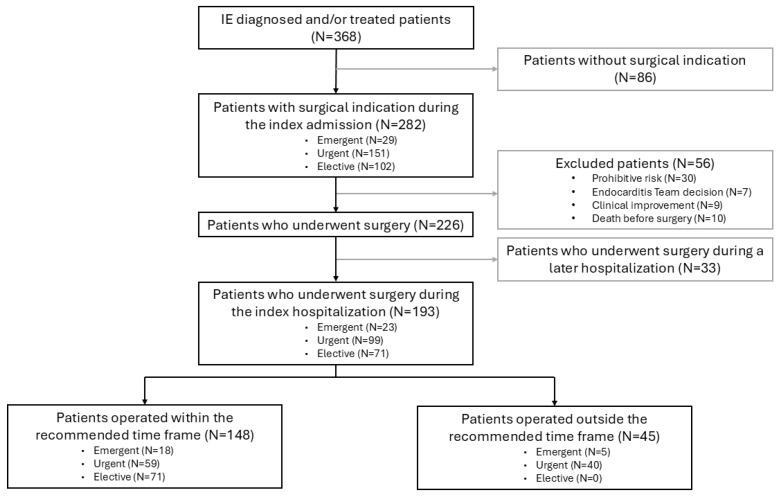
Flowchart. Abbreviations: IE: infective endocarditis.

A total of 368 patients with IE were managed during the study period; 68.5% were diagnosed at our center and 31.5% in secondary hospitals within the healthcare area, from which they were referred for treatment. Of the 282 patients (76.6%) with a surgical indication, 226 (80.1%) underwent surgery, and 193 of them (85.4%) were included in the present study after the application of the eligibility criteria. The patient flowchart is shown in Figure 1. In total, 148 (76.7%) of the included patients were operated on within the recommended time frame, and 45 (23.3%) outside the recommended time frame.

Median age was 58 years (IQR 48–70), and most patients were men (72%). The cohort presented a relevant prevalence of comorbidity, both cardiovascular and extracardiac, including the presence of cardiovascular risk factors and previous structural heart disease. Complete characteristics of the patients, according to whether surgery was performed within or outside the recommended time frame, are shown in Table 1.

To explore the potential influence of pre-referral delays on adherence to recommended surgical timing, we analyzed the interval from symptom onset to diagnosis and the proportion of patients initially diagnosed at a referring center. No significant differences were observed between patients undergoing surgery within or outside the recommended time frame in either of these variables (Supplementary Table S1).

Table 2 shows the characteristics of IE, including microbiological data and imaging findings of patients who underwent surgery, grouped according to whether surgery was performed within or outside the recommended time frame. Regarding the affected structure, 69.4% involved native valves, 23.8% involved prosthetic valves (of which, 52.2% early prosthetic valve IE), and 6.7% were CIED-related. Although there were no differences between groups in native-valve IE, a higher proportion of patients with prosthetic valve IE was observed in the group operated outside the recommended time frame (18.9% vs. 40.0%, *p* = 0.004), particularly among those with early prosthetic valve IE (6.8% vs. 31.1%, *p* < 0.001). All CIED-related IE cases underwent surgery within the recommended time frame.

According to the origin of the infection, 65.3% were acquired in the community, and 34.7% were healthcare-associated. Of these, there were differences between both groups in nosocomial origin: 8.8 vs. 20%, *p* = 0.038. The most frequently identified source of infection was dental (10.9%); however, in most cases, the source was unknown (46.1%).

Median time from symptom onset to diagnosis was 16 days (IQR 7–42.5), and median time from hospital admission to diagnosis was 1 day (IQR 1–4.5). The causative microorganism was identified in 87% of the cases, with a predominance of coagulase-negative *Staphylococcus* (20.2%). Blood cultures were negative in 13% of cases.

Altogether, 53.9% of patients had a previous diagnosis of a predisposing lesion, and after initial echocardiographic evaluation of predisposing findings, the most frequent was native valve disease (stenosis and/or regurgitation) (33.7%), followed by prosthetic valves (21.2%). The most prevalent cause of the predisposing lesion was degenerative valve disease (32.1%).

Median left ventricular ejection fraction (LVEF) was 65% (IQR 57–72.5), without differences between groups. Altogether, 90.7% had vegetations on echocardiography (transthoracic and/or transoesophageal), with the aortic valve being the most frequently affected (57%), followed by the mitral valve (46.1%).

Echocardiographic complications were described in 52.3% of the patients: valve leaflet perforation and pseudoaneurysm were the most frequent (32.6% and 11.9%, respectively). There was a higher prevalence of paravalvular abscesses and pseudoaneurysm in the group outside the recommended time frame (6.8 vs. 22.2%, *p* = 0.005 and 6.8 vs. 28.9%, *p* < 0.001, respectively), whereas valve leaflet perforation was more frequent in the within-range group (36.5 vs. 20%, *p* = 0.039). In total, 32.1% had severe mitral regurgitation and 45.1% had severe aortic regurgitation; of them, 29% and 17.2%, respectively, had previously documented valvular regurgitation.

### 3.2. Surgical Indication and Surgery During Index Hospitalization

The reason for surgical indication and the established surgical timing are shown in Table 3.

The main reason for surgical indication was HF refractory to medical treatment (46.1%), followed by uncontrolled infection (25.4%) and prevention of systemic embolism in 10.9% (in the group within the recommended time frame, 48.6%, 20.9% and 7.4%, respectively vs. 37.8%, 40% and 22.2% in those outside the recommended time frame, *p* < 0.001).

Twenty-three of the 193 patients (11.9%) had emergent priority, 99 (51.3%) had urgent priority, and 71 (36.8%) had elective priority. The distribution of surgical priority was significantly different between patients operated within and outside the recommended time frame (*p* < 0.001). Of those who underwent surgery within the recommended time frame, 18 (12.2%), 59 (39.9%), and 71 (48%) had emergent, urgent, and elective priority, respectively. Of those operated outside the recommended time frame, 5 (11.1%) and 40 (88.9%) had emergent and urgent indication, respectively, with no patients with elective indication operated outside the recommended time frame.

Different surgical time intervals were evaluated in the cohort: median time from admission to surgery was 13 days (IQR 7–24), median time from diagnosis to surgery was 10 days [IQR 5–18.5], and from surgical indication to surgery was 6 days (IQR 3–11). Observed time intervals were as expected according to the assigned priority: in patients with emergent indication, it was 1 day (IQR 1–2); in patients with urgent indication, it was 6 days [IQR 3–9], and in patients with elective indication, it was 9 days [IQR 6–15].

Preoperative surgical risk was assessed using the EuroSCORE II and Society of Thoracic Surgeons (STS) scores, and we compared it according to whether the surgery was performed within or outside the recommended time frame. According to EuroSCORE II, surgical risk was similar between both groups: 3.85 (IQR 2.16–11.77) vs. 7.12 (IQR 2.98–11.07) (*p* = 0.099). According to the STS score, statistically significant differences were found between both groups: 3.76 (IQR 1.44–8.63) vs. 6.04 (IQR 2.96–10.11) (*p* = 0.014).

Among the 56 patients with surgical indication who did not undergo surgery, 37 were not operated on due to high preoperative risk or an Endocarditis Team decision. These patients had a significantly higher STS score than surgically treated patients (9.75 [IQR 5.94–21.44] vs. 4.11 [IQR 1.70–9.06]; *p* < 0.001).

### 3.3. Thirty-Day Mortality and In-Hospital Events

**Figure 2:**
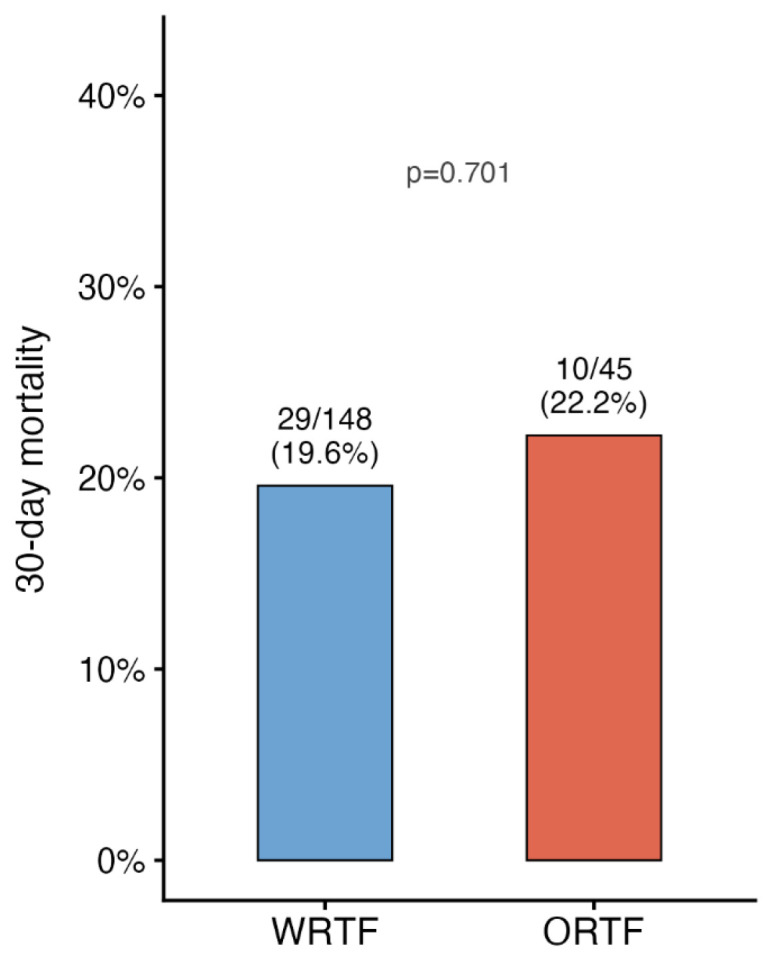
Thirty-day mortality between patients who underwent surgery within or outside the recommended time frame. Bar chart showing 30-day mortality in patients with infective endocarditis and indication for surgical treatment according to adherence to guideline-recommended surgical timing. Similar mortality was observed between patients who underwent surgery within and outside the recommended time frame. Abbreviations: ORTF: outside the recommended time frame; WRTF: within the recommended time frame.

Thirty-day mortality occurred in 39 (20.2%) of the 193 patients who underwent surgery, 29 (19.6%) in the group operated within the recommended time frame, and 10 (22.2%) in the group operated outside the recommended time frame (OR 0.85, 95% CI 0.38–1.92; *p* = 0.701) (Figure 2). The most frequent causes of death were HF (56.4%) and surgical complications (20.5%), followed by sepsis (17.9%) and CA (5.1%).

In the multivariable analysis, surgery within or outside the recommended time frame was not associated with 30-day mortality (OR 1.97, 95% CI 0.77–5.04; *p* = 0.159), after adjusting for surgical risk estimated by STS score, surgical priority, and the presence of intracardiac abscess. STS score was independently associated with mortality (OR 1.10, 95% CI 1.03–1.17; *p* = 0.003). Likewise, urgent priority showed a higher risk compared to elective priority (OR 3.36, 95% CI 1.21–9.35; *p* = 0.02), whereas emergent priority was not associated with mortality compared to elective priority (OR 1.39, 95% CI 0.29–6.59; *p* = 0.676). The presence of an intracardiac abscess showed a trend toward higher mortality, without reaching statistical significance after adjustment (OR 2.19, 95% CI 0.76–6.3; *p* = 0.147). The model showed adequate calibration (Hosmer–Lemeshow *p* = 0.16) and moderate explanatory capacity (Nagelkerke’s R^2^ = 0.183).

Additionally, a sensitivity analysis was performed in which patients with surgical indications who died before surgery were classified as belonging to the group operated outside the recommended time frame. Results were consistent with the main analysis, showing no association between surgery within the recommended time frame and 30-day mortality after adjusting for STS score, surgical priority, and the presence of intracardiac abscess (OR 1.02, 95% CI 0.46–2.27; *p* = 0.962).

During hospital admission, cardiovascular complications were frequent: 92.2% presented clinical or imaging complications, with development or worsening of HF in 38.9%, persistent bacteremia in 14.5%, embolic events in 17.6%, and echocardiographic complications without clinical significance in 21.1%, with no differences between groups. After the intervention, 63.7% developed clinical complications, HF development being the most frequent (32.1%), without differences between groups.

Regarding the New York Heart Association (NYHA) functional class at admission time, 44.6% were in advanced functional class (NYHA III–IV), with clinical signs of hemodynamic instability in 31.6% and requiring inotropic support in 29.5%. Altogether, 36.3% experienced MACE during index hospitalization, with shock as the most frequent occurrence in 21.7% of cases. There were no significant differences between groups regarding in-hospital complications and patients’ clinical status (Table 4).

### 3.4. One-Year Mortality

**Figure 3:**
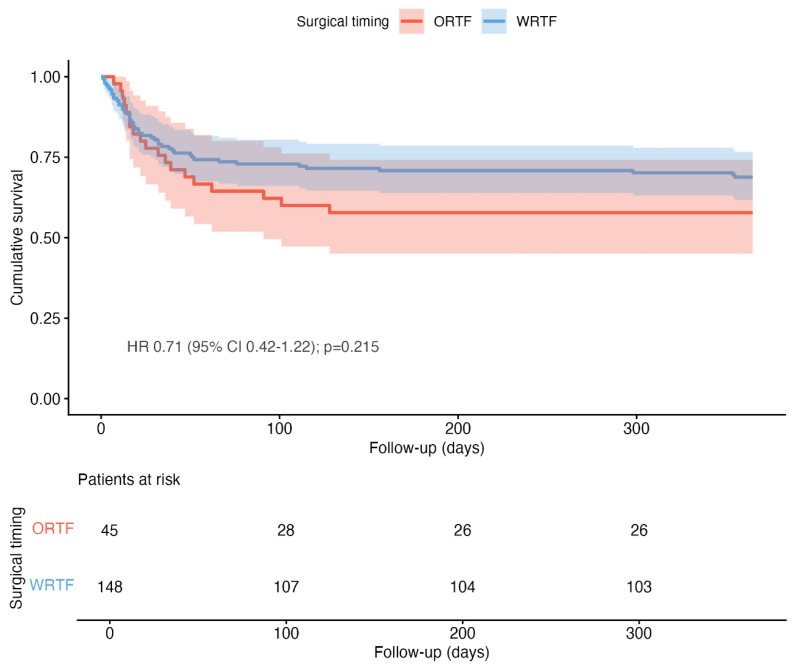
One-year survival curve in patients who underwent surgery within or outside the recommended time frame. Kaplan–Meier survival curves for 1-year survival in patients with infective endocarditis and indication for surgical treatment according to adherence to guideline-recommended surgical timing. No statistically significant differences were observed between groups. Abbreviations: ORTF: outside the recommended time frame; WRTF: within the recommended time frame.

One-year survival was 66.3% in the overall cohort, with no significant differences between patients who underwent surgery within or outside the recommended time frame in the univariable analysis (HR 0.71, 95% CI 0.42–1.22; *p* = 0.215) (Figure 3). Likewise, in the multivariable survival analysis adjusted for surgical risk estimated by STS score, surgical priority, and presence of intracardiac abscess in a Cox regression model for one-year mortality, surgery within the recommended time frame was not associated with a higher risk of death (HR 1.19, 95% CI 0.65–2.17; *p* = 0.578). STS score remained an independent predictor of mortality (HR 1.09, 95% CI 1.05–1.12; *p* < 0.001), whereas surgical priority and the presence of intracardiac abscess showed no independent association in the adjusted model (urgent priority compared to elective priority (HR 1.74, 95% CI 0.91–3.34; *p* = 0.095), emergent priority compared to elective priority (HR 0.64, 95% CI 0.23–1.80; *p* = 0.394) and intracardiac abscess (HR 1.48, 95% CI 0.73–2.99; *p* = 0.279)).

### 3.5. Sensitivity Analysis: Propensity-Score Overlap Weighting

In the propensity-score (overlap-weighted) analysis, covariate balance was achieved for all prespecified variables (all standardized mean differences <0.01; propensity score c-statistic 0.80), and the results were consistent with the absence of a survival benefit for surgery within the recommended time frame: the adjusted HR for mortality was 1.71 (95% CI 0.82–3.57; *p* = 0.155) at 30 days and 1.20 (95% CI 0.68–2.14; *p* = 0.530) at one year, consistent with the multivariable models (shown in Supplementary Figure S1).

## 4. Discussion

A substantial proportion of patients with IE require surgery as part of their treatment, with surgical indication depending on preoperative risk, which is strongly influenced by the patient’s clinical status, as well as on the potential development of complications. In this context, in the present study, we analyzed a contemporary cohort of patients with IE and surgical indication who underwent surgery during the index hospitalization over a period of more than 20 years. The aim was to evaluate whether adherence to current guideline-recommended surgical timing [17,18,19,20] was associated with patients’ prognosis.

In our cohort, no significant differences in 30-day or one-year mortality were observed between patients who underwent surgery within or outside the recommended time frame according to the established surgical priority. This finding suggests that, in patients with IE and surgical indication, strict adherence to recommended surgical timing might not constitute the main determinant of prognosis. These results remained consistent in the sensitivity analysis, which included patients with surgical indications who died before undergoing surgery, supporting the robustness of the results and suggesting that they were not exclusively driven by the exclusion of patients with a worse prognosis.

Several factors may explain the results obtained in our study. First, surgical timing in IE is not an independent exposure, but is closely influenced by disease severity, hemodynamic status, neurological complications, anatomical complexity, and individualized decision-making by the Endocarditis Team. In our study, preoperative risk (estimated using the STS score) was independently associated with short- and mid-term mortality, suggesting that it may have a stronger prognostic impact than adherence to a predefined time category. This may lead to confounding by indication in both directions: more severely ill patients may undergo earlier surgery despite a higher baseline risk of death, whereas surgery may be delayed in clinically stable but diagnostically or surgically complex patients. As a result, adherence to a predefined time category may not fully capture the appropriateness of the timing decision for an individual patient, and the potential benefit of earlier surgery may be diluted when analyzing a heterogeneous population as a whole. Therefore, surgical prioritization should probably integrate not only guideline recommendations, but also an individualized assessment of preoperative risk.

According to these findings, Song et al. [22] and Graversen et al. [23] have described that prognosis in IE is strongly influenced by clinical factors such as functional class, the presence of periannular abscesses or neurological events, as well as by surgical treatment. In this regard, patients with surgical indications who ultimately did not undergo surgery showed higher mortality rates than those who underwent surgery, despite the inherent surgical risk. Patients managed conservatively were older, had a higher burden of comorbidities, and a worse clinical status, highlighting the importance of risk assessment when considering surgical intervention.

Second, the clinical course of IE may be highly dynamic, with significant changes occurring over a short period of time. Acute complications such as valve rupture, progression of HF, or development of cardiogenic shock may rapidly modify the patient’s clinical status and, consequently, influence both surgical indication and timing. This need for individualization depends not only on the patient’s clinical evolution, but also on healthcare setting: recent studies such as that by Zulet et al. [16] have shown that, in patients referred to a reference centre for surgery, a time from diagnosis to intervention longer than seven days is associated with a higher 30-day mortality, suggesting that prolonged surgical delay may be especially relevant in scenarios with greater clinical and logistical complexity, such as referral to reference centers or high clinical complexity.

In our cohort, the interval from symptom onset to diagnosis and the proportion of patients initially diagnosed at a referring center were similar between groups, providing no clear indication that pre-referral delays were the primary determinant of differences in adherence to recommended surgical timing. Instead, patients with greater anatomical or diagnostic complexity, such as those with prosthetic valve IE or paravalvular complications, may require more extensive diagnostic work-up before a definitive surgical decision can be made, which may in turn contribute to deviations from guideline-recommended timing.

Several studies have evaluated the impact of early surgery in patients with IE. Lalani et al. [13] showed a significant mortality reduction with early surgery in native-valve IE compared with medical treatment alone, while the EASE trial [14] found that early surgery in complicated IE reduced embolic events without significant differences in mortality, in a highly selected population with strict inclusion criteria, which limits comparability with more heterogeneous cohorts such as ours. More recently, D’Alonzo et al. [24] reported that, in patients with aortic valve IE and non-emergent indication, surgery after a short course of antibiotic therapy was not associated with increased mortality or recurrence of IE compared with delayed strategies, although this finding was restricted to a specific subgroup. Taken together, these studies and our own results support the idea that the benefit of early surgery may relate more to the prevention of specific complications than to a reduction in overall mortality, and reinforce, as suggested by Kang [8], that the optimal timing of surgery should be individualized according to the risk–benefit balance, with specific situations, such as hemorrhagic CVA, potentially justifying delayed intervention despite an initial surgical indication.

Despite the available evidence, there are limited studies specifically evaluating whether adherence to recommended surgical timing is associated with prognosis in patients with IE, since most studies have focused on comparing early surgery strategies with conventional treatment or analyzing specific clinical subgroups. In this context, our findings suggest that strict adherence to these time frames might not constitute the main determinant of prognosis, which appears to be more closely related to preoperative risk and the patient’s clinical status.

Although the present study spans a long inclusion period during which clinical practice guidelines evolved, the overall framework for surgical indications and timing remains largely consistent over time. The 2023 ESC Guidelines introduced relevant updates, particularly regarding multimodality imaging and Heart Valve Centre organization; however, surgery continues to be primarily indicated for HF, uncontrolled infection, and prevention of embolic events, with timing still categorized as emergent, urgent, or elective. Therefore, although terminology and some specific recommendations have evolved, we believe that the clinical rationale underlying our classification remains applicable to current practice.

### Study Limitations

This study presents several limitations that need to be considered. First, it is an observational, retrospective, and single-center study, which may limit the generalizability of the results. Second, only patients who finally underwent surgery during the index hospitalization were included, excluding patients with surgical indications who did not undergo surgery, especially those who died before surgery. This selection might have introduced an indication bias since operated patients could present a different clinical profile in terms of severity and comorbidity. In order to explore the possible impact of this bias, we performed a sensitivity analysis including patients who died before surgery as part of the outside-the-recommended-time-frame group, representing a conservative scenario. Additionally, patients who did not undergo surgery due to high operative risk or an Endocarditis Team decision had a significantly higher preoperative risk than surgically treated patients, further supporting the presence of selection bias and limiting the generalizability of our findings to patients who ultimately underwent surgery. Another limitation is the absence of an a priori sample size calculation; the limited sample size of the outside-recommended-time-frame group may have reduced statistical power to detect modest differences between groups, and therefore, the absence of a statistically significant association should not be interpreted as evidence of equivalence. No formal correction for multiple comparisons was applied. Likewise, the broad inclusion period, which spanned more than two decades, may have influenced the results due to changes in clinical management, surgical techniques, and guideline recommendations over time, even though, as discussed above, the framework for surgical timing has remained largely stable across guideline updates. Although we adjusted the analysis for clinically relevant variables, unmeasured confounding factors cannot be ruled out.

## 5. Conclusions

In this cohort of patients with infective endocarditis and surgical indication who underwent surgery during the index hospitalization, adherence to guideline-recommended surgical timing was not associated with short- and mid-term mortality. Preoperative risk was independently associated with both short- and mid-term mortality, whereas surgical priority was associated with short-term mortality. Future prospective studies are needed to better characterize the role of surgical timing in the prognosis of infective endocarditis.

## Figures and Tables

**Table 1:** Baseline patients’ characteristics.

	Overall Cohort (N = 193)	Within Recommended Time Frame (N = 148)	Outside Recommended Time Frame (N = 45)	*p*-Value
Demographics				
Age (years)	58 [48–70]	58 [45.25–69]	61 [53.5–71.5]	0.471
Men (%)	139 (72)	104 (70.3)	35 (77.8)	0.326
Women (%)	54 (28)	44 (29.7)	10 (22.2)	
Risk factors/comorbidities				
Hypertension (%)	100 (51.8)	67 (45.3)	33 (73.3)	**0.001**
Diabetes mellitus (%)	49 (25.4)	33 (22.3)	16 (35.6)	0.074
Dyslipidemia (%)	59 (30.6)	39 (26.4)	20 (44.4)	**0.021**
Chronic kidney disease (%)	27 (14.0)	19 (12.8)	8 (17.8)	0.403
eGFR (mL/min/1.73 m^2^; MDRD-4)	75.1 [54.1–99.6]	74.85 [49.2–99.8]	75.5 [58.9–99.6]	0.268
Hemodialysis (%)	8 (4.1)	8 (5.4)	0 (0)	0.111
Stroke (%)	30 (15.5)	22 (14.9)	8 (17.8)	0.637
Immunosuppression ^1^ (%)	27 (14.0)	20 (13.5)	7 (15.6)	0.729
Intravenous drug use (%)	11 (5.7)	10 (6.8)	1 (2.2)	0.251
Previous invasive procedure (<6 months) (%)	78 (40.4)	57 (38.5)	21 (46.7)	0.329
Previous heart disease				
Previous IE (%)	7 (3.6)	6 (4.1)	1 (2.2)	0.565
Previous cardiac surgery (%)	67 (34.7)	48 (32.4)	19 (42.2)	0.227
Previous CABG (%)	4 (2.1)	2 (1.4)	2 (4.4)	0.202
History of heart failure (%)	80 (41.5)	54 (36.5)	26 (57.8)	**0.011**
History of atrial fibrillation/flutter (%)	48 (24.9)	34 (23)	14 (31.1)	0.269
History of coronary artery disease (%)	23 (11.9)	17 (11.5)	6 (13.3)	0.738
Functional class at admission				
NYHA I (%)	53 (27.5)	40 (27.0)	13 (28.9)	0.112
NYHA II (%)	54 (27.5)	47 (31.8)	7 (15.6)	
NYHA III–IV (%)	86 (44.6)	61 (41.2)	25 (55.6)	

^1^ Immunosuppression: This group included patients with active cancer, human immunodeficiency virus (HIV) infection, chronic use of immunosuppressive drugs, active autoimmune disease, and complex congenital heart disease. Abbreviations: eGFR: estimated glomerular filtration rate; IE: infective endocarditis; CABG: coronary artery bypass grafting; and NYHA: New York Heart Association.

**Table 2:** Microbiology and imaging findings.

	Overall Cohort (N = 193)	Within Recommended Time Frame (N = 148)	Outside Recommended Time Frame (N = 45)	*p*-Value
Type of IE				
Native-valve IE (%)	134 (69.4)	107 (72.3)	27 (60.0)	0.117
Prosthetic-valve IE (%)	46 (23.8)	28 (18.9)	18 (40.0)	**0.004**
Early prosthetic-valve IE (%)	24 (12.4)	10 (6.8)	14 (31.1)	**<0.001**
Late prosthetic-valve IE (%)	22 (11.4)	18 (12.2)	4 (8.8)	0.113
CIED-related IE (%)	13 (6.7)	13 (8.8)	0 (0)	**0.028**
Infection origin				
Community-acquired IE (%)	126 (65.3)	96 (64.9)	30 (66.7)	0.824
Healthcare-related IE (without admission) (%)	45 (23.3)	39 (26.4)	6 (13.3)	0.071
Nosocomial IE (%)	22 (11.4)	13 (8.8)	9 (20)	**0.038**
Source of infection				
Digestive (%)	19 (9.8)	14 (9.5)	5 (11.1)	0.362
Dental (%)	21 (10.9)	16 (10.8)	5 (11.1)	
Genitourinary (%)	13 (6.7)	11 (7.4)	2 (4.4)	
Indwelling catheter (%)	4 (2.1)	4 (2.7)	0 (0)	
Hemodialysis (%)	6 (3.1)	6 (4.1)	0 (0)	
Respiratory (%)	4 (2.1)	4 (2.7)	0 (0)	
Cutaneous (%)	3 (1.6)	1 (0.7)	2 (4.4)	
Other (%)	34 (17.6)	27 (18.2)	7 (15.6)	
Unknown (%)	89 (46.1)	65 (43.9)	24 (53.3)	
Microorganism				
Microorganism identification (%)	168 (87)	128 (86.5)	40 (88.9)	0.674
Methicillin-sensitive *Staphylococcus aureus* (%)	28 (14.5)	24 (16.2)	4 (8.9)	0.199
Methicillin-resistant *Staphylococcus aureus* (%)	12 (6.2)	9 (6.1)	3 (6.7)	
Coagulase-negative *Staphylococcus* (%)	39 (20.2)	30 (20.3)	9 (20.0)	
*Streptococcus viridans* (%)	35 (18.1)	29 (19.6)	6 (13.3)	
*Streptococcus gallolyticus* (%)	11 (5.7)	10 (6.8)	1 (2.2)	
*Enterococcus* spp. (%)	22 (11.4)	13 (8.8)	9 (20.0)	
Negative cultures (%)	25 (13)	20 (13.5)	5 (11.1)	
Others ^1^ (%)	21 (10.9)	13 (8.8)	8 (17.8)	
Echocardiography				
Known predisposing lesion (%)	104 (53.9)	78 (52.7)	26 (57.8)	0.550
Predisposing lesion ^2^ on admission echocardiography (%)	130 (67.4)	100 (67.6)	30 (66.6)	0.910
Native valve heart disease (%)	65 (33.7)	51 (35.1)	14 (31.1)	0.092
Presence of prosthetic valve (%)	41 (21.2)	27 (18.6)	14 (31.1)	
Congenital heart disease/cardiomyopathy (%)	10 (5.1)	8 (5.5)	2 (4.4)	
Presence of CIED (%)	14 (7.2)	14 (9.7)	0 (0)	
Underlying heart disease etiology (%)	115 (59.6)	86 (58.1)	29 (64.4)	0.448
Degenerative (%)	62 (32.1)	42 (28.4)	20 (44.4)	0.244
Congenital (%)	35 (18.1)	30 (20.3)	5 (11.1)	
Rheumatic (%)	15 (7.8)	12 (8.1)	3 (6.7)	
Ischaemic (%)	3 (1.6)	2 (1.4)	1 (2.2)	
LVEF (%)	65 [57–72.5]	62.5 [55.75–72.25]	64 [57–70]	0.313
LVEF ≥ 50% (%)	181 (93.8)	139 (93.9)	42 (93.3)	0.887
LVEF < 50% (%)	12 (6.2)	9 (6.1)	3 (6.7)	
Presence of vegetations (%)	175 (90.7)	134 (90.5)	41 (91.1)	0.587
Vegetations on TTE (%)	140 (72.5)	108 (73)	32 (71.1)	0.806
Vegetations on TOE (%)	145 (75.1)	110 (74.3)	35 (77.8)	0.802
Location of vegetations				
Mitral location (%)	89 (46.1)	68 (45.9)	21 (46.7)	0.932
Aortic location (%)	110 (57)	79 (53.4)	31 (68.9)	0.066
Mitral–aortic location (%)	23 (11.9)	14 (9.5)	9 (20.0)	0.056
Tricuspid location (%)	9 (4.7)	9 (6.1)	0 (0)	0.086
Pulmonary location (%)	3 (1.6)	2 (1.4)	1 (2.2)	0.551
CIED lead location (%)	13 (6.7)	13 (8.8)	0 (0)	**0.028**
Presence of significant valvular disease/echocardiographic complications				
Presence of significant MR (%)	62 (32.1)	52 (35.1)	10 (22.2)	0.104
Presence of significant AR (%)	87 (45.1)	67 (45.3)	20 (44.4)	0.922
Presence of echocardiographic complication (%)	101 (52.3)	81 (54.7)	20 (44.4)	0.226
Valve leaflet perforation (%)	63 (32.6)	54 (36.5)	9 (20.0)	**0.039**
Pseudoaneurysm (%)	23 (11.9)	10 (6.8)	13 (28.9)	**<0.001**
Intracardiac abscess (%)	20 (10.4)	10 (6.8)	10 (22.2)	**0.005**
Prosthetic valve dehiscence (%)	12 (6.2)	8 (5.4)	4 (8.9)	0.296
Fistula (%)	6 (3.1)	4 (2.7)	2 (4.4)	0.427
Valve aneurysm (%)	2 (1.0)	2 (1.4)	0 (0)	0.587

^1^ Others: Fungal, polymicrobial, *Streptococcus pneumoniae*, *Coxiella burnetii*, *Pseudomonas aeruginosa*, *Streptococcus agalactiae*, HACEK group, and atypical microorganisms. ^2^ Predisposing lesions: Native valve disease (including mitral stenosis and/or regurgitation, aortic stenosis and/or regurgitation and bicuspid aortic valve), presence of prosthetic valve, congenital heart disease/cardiomyopathy (including complex congenital heart disease and hypertrophic cardiomyopathy), and intracardiac electronic devices. Abbreviations: IE: infective endocarditis; CIED: cardiac implantable electronic device; TTE: transthoracic echocardiography; TOE: transoesophageal echocardiography; LVEF: left ventricular ejection fraction; MR: mitral regurgitation; and AR: aortic regurgitation.

**Table 3:** Surgical indications and surgical priority.

	Overall Cohort (N = 193)	Within Recommended Time Frame (N = 148)	Outside Recommended Time Frame (N = 45)	*p*-Value
Reason for surgical indication				
Heart failure (%)	89 (46.1)	72 (48.6)	17 (37.8)	**<0.001**
Uncontrolled infection (%)	49 (25.4)	31 (20.9)	18 (40.0)	
Prevention of embolic events (%)	21 (10.9)	11 (7.4)	10 (22.2)	
CIED infection (%)	12 (6.2)	12 (8.1)	0 (0)	
Other indications (elective) (%)	22 (11.4)	22 (14.9)	0 (0)	
Surgical priority				
Emergent (<24 h) (%)	23 (11.9)	18 (12.2)	5 (11.1)	**<0.001**
Urgent (<7 days) (%)	99 (51.3)	59 (39.9)	40 (88.9)	
Elective (≥7 days) (%)	71 (36.8)	71 (48)	0 (0)	

**Table 4:** In-hospital complications and clinical evolution.

	Overall Cohort (N = 193)	Within Recommended Time Frame (N = 148)	Outside Recommended Time Frame (N = 45)	*p*-Value
IE-related complications (%)	178 (92.2)	134 (90.5)	44 (97.8)	0.094
De novo heart failure (%)	54 (28.0)	41 (27.7)	13 (28.9)	0.311
Worsening heart failure (%)	21 (10.9)	16 (10.8)	5 (11.1)	
Persistent bacteremia (%)	28 (14.5)	25 (16.9)	3 (6.7)	
Systemic embolism (%)	34 (17.6)	25 (16.9)	9 (20.0)	
Asymptomatic echocardiographic complication (%)	41 (21.2)	27 (14.0)	14 (31.1)	
MACE before surgery ^1^ (%)	70 (36.3)	51 (34.5)	19 (42.2)	0.343
Stroke (%)	16 (8.3)	14 (9.5)	2 (4.4)	0.325
Intracranial hemorrhage (%)	4 (2.1)	3 (2.0)	1 (2.2)	
Acute myocardial infarction (%)	1 (0.5)	1 (0.7)	0 (0)	
Shock (%)	42 (21.7)	30 (20.1)	12 (26.7)	
Cardiac arrest (VT/VF) (%)	4 (2.1)	3 (2.0)	1 (2.2)	
Pulmonary thromboembolism (%)	3 (1.6)	2 (1.4)	1 (2.2)	
Postoperative complications ^2^				
Postoperative complications (%)	123 (63.7)	92 (62.2)	31 (68.9)	0.411
Postoperative cardiac arrest (VT/VF) (%)	14 (7.2)	11 (7.6)	3 (6.7)	0.581
Postoperative stroke (%)	7 (3.6)	5 (3.4)	2 (4.4)	0.515
Postoperative major bleeding (%)	31 (16.1)	21 (14.2)	10 (22.2)	0.199
Postoperative heart failure (%)	62 (32.1)	46 (31.1)	16 (35.6)	0.574
Postoperative shock				
Hemorrhagic shock (%)	17 (8.8)	11 (7.4)	6 (13.3)	0.588
Septic shock (%)	29 (15)	24 (16.2)	5 (11.1)	
Distributive shock (%)	2 (1.0)	1 (0.7)	1 (2.2)	
Cardiogenic shock (%)	29 (15)	22 (14.9)	7 (15.6)	

^1^ No cases of major bleeding as preoperative MACE were recorded in either group. ^2^ No cases of pulmonary thromboembolism were recorded after surgery. Abbreviations: IE: infective endocarditis; VT: ventricular tachycardia; VF: ventricular fibrillation; and MACE: major adverse cardiovascular events.

## Data Availability

The data presented in this study are available from the corresponding author upon reasonable request. The data are not publicly available due to ethical and privacy restrictions.

## Supplementary Materials

The following supporting information can be downloaded at https://www.mdpi.com/article/10.3390/jcm15145421/s1. Figure S1: Covariate balance before and after propensity-score overlap weighting (ATO). Standardized mean differences for all covariates fall within the 0.10 threshold (dashed lines) after weighting, indicating excellent covariate balance; Table S1: Available variables related to the pre-referral period according to adherence to the guideline-recommended surgical time frame.
