# Supplementary material for: Impact of Adherence to Guideline-Recommended Surgical Timing on Outcomes in Infective Endocarditis
Source: J Clin Med. 2026 Jul 10;15(14):5421. doi: 10.3390/jcm15145421 (PMC13412765; doi:10.3390/jcm15145421)
Supplement: Supplementary file 1 [file jcm-15-05421-s001.zip › jcm-4389583-supplementary.pdf]

Table S1. Available variables related to the pre-referral period according to adherence to the guideline-recommended surgical time frame.

	Overall cohort (N=193)	Within recommended time frame (N=148)	Outside recommended time frame (N=45)	*p-value*
Time from symptom onset to diagnosis (days)	16 [7-42.5]	16 [7-43.8]	21 [7-42.5]	0.710
Patients diagnosed at reference centre (%)	124 (64.2)	99 (66.9)	25 (55.6)	0.165
Patients diagnosed at referring hospital (%)	69 (35.8)	49 (33.1)	20 (44.4)	

Data are presented as median [interquartile range] or n (%).

Figure S1. Covariate balance before and after propensity-score overlap weighting.

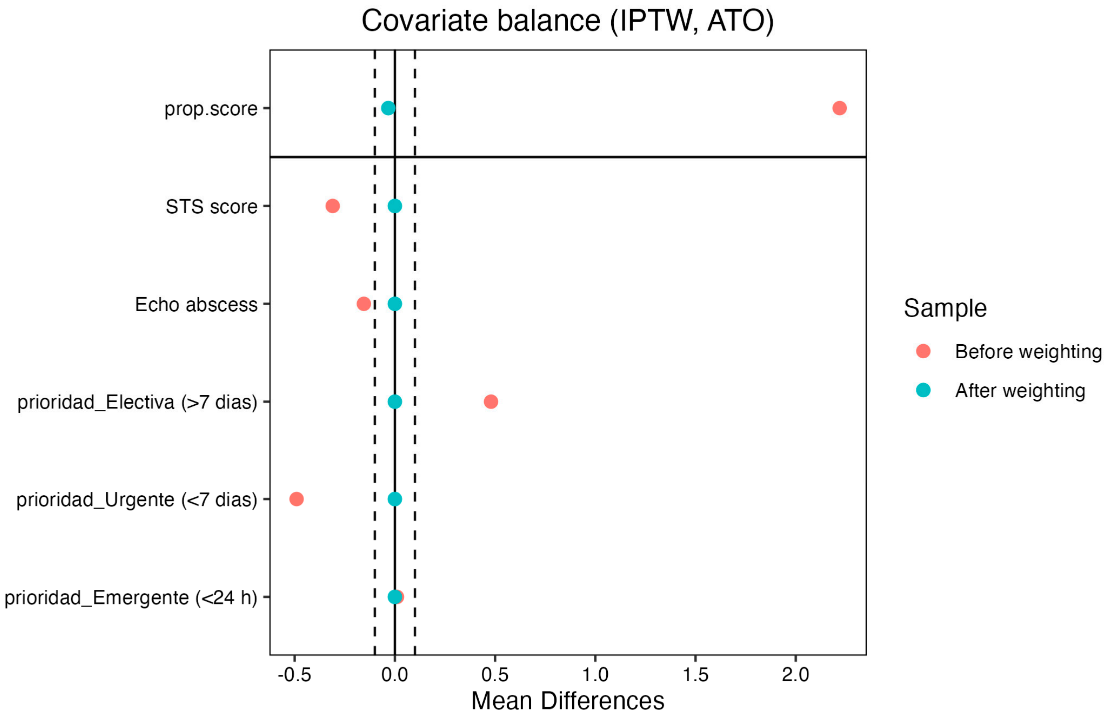

Figure S1. Covariate balance before and after propensity-score overlap weighting (ATO). Standardized mean differences for all covariates fall within the 0.10 threshold (dashed lines) after weighting, indicating excellent covariate balance.
